# Exploring the mental health of young people in households and schools in Gorongosa District, Center of Mozambique

**DOI:** 10.1038/s41598-024-79257-7

**Published:** 2024-11-14

**Authors:** Victor Igreja, Taryn Axelsen, Alana Brekelmans

**Affiliations:** 1 School of Humanities and Communication, University of Southern Queensland, Springfield Campus, Springfield Central, QLD 4300 Australia; 2 School of Mathematics, Physics and Computing, University of Southern Queensland, Springfield Campus, Springfield Central, Australia; 3 Faculty of Business, Economics and Law, The University of Queensland, St. Lucia Campus, Brisbane, Australia

**Keywords:** Young people, Depression, Anxiety, Sleeping disturbances, Risks and protective factors, Rural settings, Mozambique, Health care, Risk factors, Signs and symptoms

## Abstract

The mental health needs of young people in sub-Saharan African societies remain understudied. This study builds upon the everyday perspectives of young people in Gorongosa, a rural district in central Mozambique, to determine the frequency and severity of key mental health issues, identify significant risk and protective factors, and their associations with demographic factors and mental health predictors. This cross-sectional study gathered culturally and ecologically relevant variables, such as sociodemographic indicators and risk and protective factors. The Beck Depression Inventory-II (BDI-II), Self-Report Questionnaire (SRQ) and Nocturnal Intrusions after Traumatic Experiences Questionnaire (NITE) were used to determine the prevalence and severity of key mental health issues. A convenience sampling was used involving *n* = 794 young people of those (45.8%) were females and the mean age of all participants was 17.24 years (SD 2.9). To examine the results, univariate analysis, chi-square tests, binary logistic regression analyses and odds ratio were used. Risk factors were pervasive in households, communities, and schools. Mental health problems were commonly linked to severe depression (14.2%), anxiety-related disorders (39.3%), sleep disturbances (37.3%), and anxiety dreams (27.5%). Gender, age, and protective factors were associated with the frequency and severity of mental health outcomes, while living with both parents protected against the severity of anxiety-related disorders. Understanding the contextual mental health needs of young people in rural Mozambique is crucial for developing insights to craft and implement targeted public mental health interventions in resource-limited settings. Addressing mental health issues among young people necessitates a concentrated effort on understanding and managing the interplay of risks and protective factors within families, communities, and schools.

## Introduction

The mental health needs of young people in sub-Saharan African societies remain understudied. The lack of studies of common mental health problems such as mood disorders, sleeping problems, and anxiety dreams, is further acute in Lusophone Africa, also known as the Portuguese-Language Countries’ Community. These conditions are aggravated by the inability of the state authorities to implement social services over the entire national territories let alone provide mental health services and qualified professionals to assist the populations. This predicament constitutes a serious limitation for evidence-based planning of public mental health priorities and interventions tailored for young people. This article addresses this gap by examining the prevalence and severity of common mental health problems among young people in Mozambique, a Lusophone country, that has experienced past conflicts and continues to face significant challenges such as unbridled poverty, high unemployment, as well as domestic, community and political violence. These conditions constitute serious risks for young people’s mental health^1–5^.

Studies of mental health problems of young people in sub-Saharan Africa have often been conducted either among the generations directly exposed to civil war violence^3–11^ or in Anglophone and Francophone countries that did not experience postcolonial civil wars but have been exposed to poverty, unemployment, and chronic insecurity with detrimental consequences for young people’s mental health^12–15^. Rates of mental health problems (e.g., depression, anxiety, sleeping problems and nightmares) among young people vary in the continent. A study of university students in Ghana determined an incidence of 31.1% for mild to moderate depression while 8.1% reported severe depression symptoms^12^. Another study examined the incidence of depression in five East African countries and detected symptoms of depression among the studied sample as follows 64% (Tanzania), 48% (Malawi), 38% (Zimbabwe), 37% (Kenya), and 33% in the Zambian sample^13^. A comparative study involving samples of young people that had experienced forced sexual intercourse (FSI) and those that had not experienced it showed that among young people that experienced FSI, the percentages of moderate to serious mental distress were 54% (Uganda), 52% (Zambia), and 33% in Nigeria^14^. Still, another study of adolescent girls and young women in-school and out of school in Tanzania revealed a 36% incidence of depression and 31% of anxiety among out of school participants, while 6% of the sample had severe symptoms of anxiety and depression^15^. Although these studies used different diagnostic instruments, they nevertheless present important results and offer meaningful insights to probe in other contexts and social circumstances: Sex predicts the incidence of depression and anxiety disorders, with young women consistently having higher rates than males^16–18^ while the relation between incidence of depression and anxiety and age groups is less conclusive with studies suggesting a higher incidence in aged people when compared to younger people^19,20^. Other studies have indicated that the age group starting in early teens (15–64 years) appears the most impacted in cross-national samples^21^, followed by the age group (0–14 years)^16^. Mood disorders left undiagnosed and untreated can lead to detrimental outcomes. Most mental disorders have their onset during the adolescence period^22,23^ including the risk of suicides among elementary school-aged children (ages 5–12 years)^24^. Thus, the need to focus on depressive and anxiety disorders among young people, while controlling for gender and age should constitute an analytic priority^1,2,25,26^. Furthermore, despite the existing knowledge, there is still a marked shortage of analysis on sleeping disturbances and anxiety dreams facing young people in sub-Saharan Africa^5^.

A comprehensive analysis of the incidence of mood disorders should also probe into the dynamics of risk factors and potential beliefs and practices that might function as protective factors^27^. There are risk factors for mental health problems that have been recurrently identified across cultures (e.g., chronic poverty, alcohol and drug abuse, social instability and violence, sexual harassment, sexual violence and excessive exposure to social media). Yet, the diversity of contexts and conditions in which young people grow up suggests the need to consider the specific roles of socio-cultural, political, and economic contexts in the analysis of risk factors^2,28–30^. It is generally recognized that protective factors play a role in mitigating the effects of risk factors. What needs further analysis and knowledge is the content and dynamic of such risks and protections in specific contexts and social circumstances^31^. For that this study included the perceptions and voices of rural young people during the pilot stages of the research to map their views on the potential risks and protective factors existing in their social milieu at family, community, and school levels. At a further stage of the research, such factors were used to investigate in a larger sample through quantitative survey.

The study of the mental health of young people in Mozambique is still in its infancy. Recent cross-sectional studies undertaken in specific regions of the country focused on depression and anxiety among adult populations^32,33^, female heads-of-household^34^, women as victims and perpetrators of intimate partner violence and concurrent outcomes on depression, anxiety, and somatization^35^. A rare study that focused on in-school adolescents analysed the prevalence of suicidal ideation, suicidal plans, and suicidal attempts^36^. Previous qualitative and quantitative studies of the various impacts of the civil war (1976–1992) and war traumas were extensively conducted in the Gorongosa, a rural district in the centre of Mozambique^37,38^ involving mixed samples of adults and young people, and some trauma-focused interventions were applied to support the mental health recovery of the wartime generations^39^. However, this was the first study in the region that specifically focused on young people who were born after the end of the civil war. As part of the post-civil war reconstruction and development efforts, the central government has incentivized agriculture and rebuilt new markets, schools, and health posts. Yet, most people are still confronted with daily stressors^40–42^ such as unbridled poverty, unemployment, and domestic and community violence, which are known to be risks for young people. This study builds upon the everyday perspectives of young people in Gorongosa to (i) determine the prevalence and severity of key mental health issues, (ii) identify significant risk and protective factors, (iii) identify associations with demographic factors, (iv) identify impact of risk and protective factors on mental health, and (v) analyse mental health predictors.

## Methods

### Study design

**Pilot study phase 1**: In January 2012, we conducted the first local pilot study to gather culturally and ecologically relevant variables, such as sociodemographic indicators and risk and protective factors. This phase involved individual and group interviews with young people (*n* = 25), and five focus-group discussions with primary and secondary school teachers (*n* = 30), which provided insights about the mental health problems the participants considered as common to the local young people, the factors they regarded as either promoting or hindering psychological functioning. We conducted content and thematic analysis of the interviews to draw out emergent themes related to mental health problems, risks and protective factors that were consistent across respondents. **Pilot study phase 2**: In February 2012, we undertook the second pilot testing to adapt and test the quantitative scales. We used a sample of *n* = 20 participants to assess item clarity, scale length, and comprehensibility. We used the feedback to refine the items and increase clarity. When we reached saturation, we compiled the last versions of semi-structured questionnaire that comprised of demographic data and risk and protective factors, and common mental health problems. **The main study**: Upon completion of the two pilot studies, the full-scale survey was then applied in the larger sample in 2013 in households and two schools.

### Setting and ethics clearance

The general population census has established that around 176,845 people dwelled in the Gorongosa region, and the people rely mostly on agriculture for self-sustenance. Since the end of the civil war (October 1992) and following the government’s post-civil war reconstruction efforts, districts such as Gorongosa were removed from the isolation of the initial peace years, which facilitated the reestablishment of manual agricultural and artisanal mining activities and consumption of mass media technologies. Yet, unbridled poverty, unemployment, and domestic, community and political violence continue to pose significant challenges both in the region and across the country. These conditions have been further aggravated as the provision of mental health services and assistance has not been a government priority. According to some of the socio-cultural features of this region, collective orientation, and parental guardianship (known in the local language as *ku himirira*) remain central to family organization and maintenance. However, children as young as six are often expected to take on adult responsibilities, such as caring for younger siblings or tending to the sick)^42^. Furthermore, during the country’s civil war, the belligerents declared that the conflict spared no age. These disturbances from the civil war had lasting impacts on family dynamics. Since the conflict ended, even children as young as eight have been required to represent themselves in community courts to address various interpersonal conflicts^43^.

To undertake this study, the Ethics Board of the University of Queensland approved the research project (Clearance No: 2009001021, date—01/01/2012) as part of the University of Queensland Earlier Career Research Grant (2009–2012). Other funding support was provided by the Netherlands Organization for Scientific Research (NWO) and hosted at the Leiden University Medical Center (LUMC), and the Volkswagen Foundation (Hanover, Germany) and hosted by the University of Hamburg. Informed consent was obtained through the local government authorities, which the first author has had research collaboration since 1996. Informed consent was also obtained from the schools’ headmasters in Gorongosa district to involve the young people in the research at the school levels. Permission was also obtained from the legal guardians of young people to involve them as research participants in their households. All methods, including the presentation of supplementary information, were performed in accordance with the Declaration of Helsinki.

### Participants

A convenience sampling guided the selection of the participants. School settings constituted the most practical way to identify and select young participants. To minimize selection bias, we extended the selection sites to include households to reach out young respondents that were not attending schools. According to some of the local cultural beliefs and practices, age is not determined biologically. We considered these cultural norms, but we also set the eligibility criteria to be the ability to read and write and fill in the questionnaire individually or as interviewed. Thus, we enrolled young people as culturally defined with ages ranging between 9 and 21 years. This age range was also consistent with the age groups within the school system namely pre-secondary (G1, 9–15 yrs), secondary (G2, 16–19 yrs), and pre-university (G3, 20–21 yrs). The mean age of all participants was 17.24 years (SD 2.9).

### Variables

Demographic characteristics consisted of: Gender, age, marital status, profession, residential status, parental relationship, and parental status. The risk factors were organized in three categories involving the family, community, and school, which comprised 16 questions to be answered in “Yes” or “No” format. Risks in the family (7 items) - “Beaten at home”, “fight at home”, “crying at home”, “insulted at home”, “going to school without eating [food insufficiency]”, “alcohol consumption at home”, and “perceptions of quality of family relations.” Risks in the community (4 items) - “Attacked while going to school”, “Sexual assault while going to school”, “Fear of attack” and “fear of something.” Risks at school (5 items) - “Insulted in the school”, “notebook stollen”, “text stollen”, “beaten at school” and “forced to have sex at school.”

The protective factors were “parents live together/separate”, “number of siblings”, “possession of a radio at home”, and “possession of television at home”, “working besides study” and “participation in church.”

For risk and protective factors, participants received a score of 1 for “Yes” responses and 0 for “No” response. Participants who did not respond or the question was not applicable were treated as missing data.

### Mental health scales

Following the pilot studies, the large quantitative data gathering unfolded in 2012. Most studies using the DBI-II and SRQ-20 were validated and conducted with adolescents older than 12 years old. In this study, we validated the DBI-II and SRQ-20 with young people as culturally defined (from 9 years old). In relation to the NITE, previous studies had only been validated with adults. This was the first time that we applied the questionnaire among this study group. Interestingly, the NITE was the instrument that required fewer adaptations in terms of content validation (compared to the DBI-II and SRQ-20) because in Gorongosa, sleeping patterns, dreams and nightmares have more impact on the way people think about and organise their lives and plan the future than in Western societies (38,44).

The BDI-II (23 items)^44^ was designed to capture psychological and somatic symptoms manifested within a 2-week period. The instrument has been previously used in various empirical studies of depression involving adolescents in Western and non-Western societies as well as reviews and meta-analysis to determine its psychometric properties. Various studies have shown high levels of internal consistency of BDI-II. For example, in a study of a multiethnic population of young people in the United States, the coefficient alpha was 0.89^45^; in a study of Portuguese young students, the Cronbach’s coefficient α was 0.90^46^; in a study of Hong-Kong adolescents, the Cronbach’s coefficient α was 0.94^47^. Furthermore, in a study of HIV-positive adolescents in Malawi, the Cronbach’s coefficient α was 0.80^26^, while a study of young South Africans showed a DBI-II internal consistency of 0.84^48^. The BDI-II had never been validated with young children in this region. For that, we conducted two pilot studies to adapt the BDI-II to the local context, and it was administered as a self-report screening measure. The adaption consisted first in translating the BDI-II from English into Portuguese and then into the local language (Chi-Gorongose) by bilingual members from Gorongosa. The translations were then translated back into Portuguese by different interpreters to establish the linguistic equivalents for each of the items on the questionnaire. This validation process determined that the response format in the original BDI-II was not easily understood by participants. Consequently, the response scale of the BDI-II was truncated into a 3-point scale: “no” (scored 0), “sometimes” (scored 1) and “often” (scored 2). Based on pilot testing, two questions in the original instrument were re-worded and each divided into two questions. For instance, the question regarding “changes in sleeping pattern” was divided into two questions: “Do you have difficulty falling asleep?” and “Do you have difficulty staying asleep?” Furthermore, the question regarding “changes in appetite” was divided into two questions: “Do you lack appetite?” and “Do you crave food all the time?” As a result, the locally adapted version of the BDI-II comprised 23 questions. Scores were aggregated such that the maximum total score was 46. Because of the changes to the items and endpoints, no clinical cut-offs were applied, and the scale was interpreted as a continuous measure of depression symptoms. Cronbach’s alpha for this 23-item measure was strong (α = 0.917).

The Self-Report Questionnaire (SRQ-25) is a tool designed by the World Health Organization to screen for common anxiety-related disorders in developing countries^49,50^. The sub-set part of 20 questions has demonstrated high content validity with neurosis, depression, anxiety, and psychosomatic complaints^23,49,50^. The SRQ-20 has been widely used in studies of mental health problems in both conflict and non-conflict societies involving young people^23,51–53^. The SRQ items are scored 0 (‘no’, symptoms absent) or 1 (‘yes’, symptoms present) and the item scores are summed to obtain a total score. Previous studies conducted in Gorongosa using the SRQ-20 validated this scale^37–39,54^. To apply for younger children in this study, the validation process during the pilot studies revealed that one question (“do you have poor digestion?”) was found confusing due to experience with food poisoning in the two schools, and so was dropped from analysis. Thus, the scores for this measure ranged from a minimum of 0 to a maximum of 20, and the cut-off points recommended by the WHO of 40% or more positive answers to screen cases was applied. This determination of cut-off point scores has been applied in numerous studies with young people^23,55,56^. Cronbach’s alpha for the scale indicated satisfactory reliability (α = 0.929).

Sleep disturbances and nightmare experiences were assessed using the Nocturnal Intrusions after Traumatic Experiences Questionnaire (NITE)^38,56^ which was designed to assess whether individuals experienced disruptions to sleep patterns, anxiety-related dreams, post-traumatic nightmares, and accompanying physical reactions study over the previous four weeks. NITE has shown the ability to capture sleep disturbances and nightmare experiences related to any type of traumatic events^57,58^. If a respondent states that he or she did not have a nightmare in the past four weeks, the questionnaire proceeds to questions related to disruptions to sleep patterns (e.g., “In the past four weeks, did something make you sleep bad?” NITE has been applied among clinical and population samples of adults across war and post-war generations including previously in this region^37–39^. The validation process consisted in establishing the content and linguistic equivalents among this cohort. This study was the first to adapt and apply the NITE among young people in this region. NITE has shown the ability to capture sleep disturbances and nightmare experiences related to any type of traumatic events^38,57,58^, while categorizing post-traumatic nightmares as replicative (closely replicates a traumatic event), symbolic (reflects some traumatic event elements but less replicative), or mixed (a combination of replicative and symbolic)^38^. NITE scores can be analyzed in either a qualitative or quantitative fashion; it is a two-part (partially) structured questionnaire that contains 14 quantitative items and 3 qualitative ones. Only 14 quantitative items were used, with a focus on general sleeping problems and the presence, content, and impact of anxiety dreams on respondents. NITE-14, items are scored 0 (‘no’, symptoms absent) or 1 (‘yes’, symptoms present). In this case, the scores were averaged such that the overall score ranged from 0 to 1. Cronbach’s alpha for this 14-item measure was strong (α = 0.943).

For the community-based survey (*n* = 595, 74.9%), research assistants administered the questionnaire in the form of a semi-structured interview, whereas in the schools (*n* = 199, 25.1%) the participants answered the questionnaires individually as self-reports. Since the class sizes were large, we divided the students into groups so that each student had a single chair and desk, and the research assistants oversaw the process in the classroom without input or interference from classroom teachers, which ensured that participants had confidentiality.

### Statistical analysis

All results were examined using IBM SPSS Statistics (Version 27). Preliminary univariate analysis was completed on all variables to ensure consistency and to address the first aim of this report. As stated above, the internal consistency was excellent. To address the second aim, the chi-square test of independence was run on all the baseline variables (demographics, risk, and protective measures) to assess for group differences in the symptoms of BDI-II, SRQ, and NITE. This allowed us to ascertain the associations between the baseline variables and the frequency and severity of mental health issues in our sample. Exploratory binary logistic regression analyses were performed to ascertain which variables contribute most significantly to the prediction of mental health problems (BDI-II, SRQ and NITE) in the participants. All three questionnaires were converted to a binary form for this section of the analysis, to allow for ease of interpretation (mental health problems present or not present), with these being the outcome variable. Demographic variables, risk, and protective factors were added into the model as predictors. Each of the aggregated scale of the risk factors provided a cumulative measure of risk within each domain. The higher scores (50% and above) in each domain indicated greater exposure to adverse experiences. Cronbach’s alpha was calculated for each of the aggregated scales (Family Risk 0.725, School Risk 0.823, Community Risk 0.66) to determine internal consistency. Similarly, protective factors with higher scores in each domain indicated greater protection. Odds ratios were examined for those predictors that were significant in predicting the presence of mental health problems among the research participants and to allow ease of interpretation.

## Results

### Participants

The total research participants were composed of *n* = 794 of those *n* = 364 (45.8%) were females (Table 1). Participants recruited through the household survey were *n* = 595 (74.9%) and in two schools, a secular, and a Catholic school (grades 6–10) (*n* = 199, 25.1%). Most respondents were students *n* = 634 (79.8%), while the remaining approximately 20% were involved in some form of informal employment. Of the students, there were pre-university students *n* = 429 (54%), secondary school *n* = 167 (21%), and primary school *n* = 45 (5.7%). Most of the participants were unmarried *n* = 770 (97%) and only *n* = 23 (2.9%) declared to be married. The residence status of the participants was as follows: *n* = 272 (34.3%) lived with relatives other than the biological parents; *n* = 217 (27.3%) lived with both parents (father and mother); *n* = 164 (20.7%) lived with a partner; *n* = 95 (12%) lived with the mother; *n* = 37 (4.7%) lived alone; and *n* = 9 (1.1%) lived with the father.

**Table 1:** Demographics and incidence of depression, anxiety-related disorders, and sleep disturbances and anxiety dreams.

Variables	*N* = 794 (Fr.)	Percentage (%)
Gender		
Male	430	54.2
Female	364	45.8
Age		
G1: 9–15 years	158	19.9
G2: 16–19 years	455	57.3
G3: 20–21 years	181	22.8
Marital status		
Single	770	97.0
Married	23	2.9
Other	1	0.1
Living parents		
Both	530	66.8
None	127	16.0
Mother only	107	13.5
Father only	27	3.4
Siblings		
4 or less	327	41.2
5 or less	467	58.8
Profession		
School	734	79.8
Working	160	20.2
School level		
Primary	45	5.7
Secondary	167	21.0
Pre-university	429	54.0
Not at School	153	19.3
Depression (BDI-II)		
None	364	45.8
Mild	176	22.2
Moderate	141	17.8
Severe	113	14.2
Anxiety-related disorders (SRQ)		
Low or no symptoms	482	60.7
Symptoms present	312	39.3
Sleep disturbances and anxiety dreams (NITE)		
None	280	35.3
Some	218	27.5
Severe	296	37.3

Respondents’ parental relations were as follows (Table 1): *n* = 530 (66.8%) stated that they had living parents, while *n* = 127 (16%) stated that they had no living parents. Among those with parents, *n* = 107 (13.5%) stated that only the mother was alive while *n* = 27 (3.4%) only had a father. For those with living parents, *n* = 433 (54.5%) of the participants stated their parents lived together, while *n* = 354 (44.6%) reported that the parents did not live together. In relation to siblings, *n* = 467 (58.8%) stated that they had five or more siblings, while *n* = 327 (41.2%) had four or less.

### Symptoms of BDI-II, SRQ and NITE and risk and protective factors

Table 1 shows that 17.8% (*n* = 141) had moderate depression, 22.2% (*n* = 176) had mild depression, while 14.2% (*n* = 113) of the participants had severe depression. Anxiety-related disorders (SRQ-20) were identified in 39.3% (*n* = 312) of participants. Severe sleeping problems and anxiety dreams were reported by 37.3% (*n* = 296), while 27.5% (*n* = 218) reported some nightmare symptoms. Table 2 shows the percentages of risk and protective factors. The risks with the highest percentage in the home space were “going to school without food” (74.8%), “insulted at home” (60.8%), and “beaten at home” (50.1%). In the community space, the risks were “afraid of being attacked” (81.6%) and “fear of something” (76.7%) while 38.2% revealed experiences of being attacked on the way to school. In the school space, the risks that were identified with the highest percentages were “being insulted” (45.1%), “notebooks stolen” (37.9), “textbook stolen” (32.2%), and “bullied at school” (32.7%). Table 2 also presents the results of factors which the participants considered protective namely “church participation” (69.8%), having “five or less” siblings (58.8%), “parents live together” (54.5%), “having a radio at home” (53.7%).

**Table 2:** Risk and protective factors.

Risk factors	*N* = 794 (Fr.)	Percentage (%)	Protective factors	*N* = 794 (Fr.)	Percentage (%)
Risks in the family			Protective factors		
Beaten at home			No. of siblings		
No	396	49.9	4 or less	327	41.2
Yes	398	50.1	5 or less	467	58.8
Parents fight at home			Work status		
No	460	57.9	No	688	86.6
Yes	334	42.1	Yes	106	13.4
Cry at home			Parents live together		
No	420	52.9	No	354	44.6
Yes	374	47.1	Yes	433	54.5
Insulted at home			Live with		
No	311	39.1	Father	9	1.1
Yes	483	60.8	Alone	37	4.7
			Mother	95	12.0
			Partner	164	20.7
			Other	272	34.5
Going to school without food			Church participation		
No	200	25.2	No	240	46.3
Yes	594	74.8	Yes	554	69.8
Parents drink alcohol			Radio in house		
No	440	55.4	No	368	46.3
Yes	354	44.6	Yes	426	53.7
Relationship with parents					
Good	477	60.1			
Bad	317	39.9			
Risks in the community					
Attacked on way to school					
No	491	61.8			
Yes	303	38.2			
Sexually assaulted on way to school					
No	672	84.6			
Yes	122	15.4			
Afraid of being attacked					
No	146	18.4			
Yes	648	81.6			
Fear of something					
No	185	23.3			
Yes	609	76.7			
Risks at school					
Insulted at school					
No	436	54.9			
Yes	358	45.1			
Notebooks stolen					
No	493	62.1			
Yes	301	37.9			
Textbook stolen					
No	538	67.8			
Yes	256	32.2			
Bullied at school					
No	534	67.3			
Yes	260	32.7			
Forced to have intercourse					
No	782	98.5			
Yes	12	1.5			

### Associations of gender, age, risk factors and depression

Table 3 shows the association between gender, age, risk factors, and depression (DBI-II) using the chi-square test of independence. The results were significant (χ^2^ (3) = 21.713, *p* < 0.0001) to indicate an association between gender and depression symptoms (DBI). Male participants were more likely (50%) to have no depression symptoms present in comparison with female participants (40.9%). Whereas female participants were more likely to have severe depressive symptoms (20.3%) in comparison with males (9.1%). In relation to the association between age categories (9–15; 16–19; 20–21) and depression (DBI-II) (Table 3), the chi-square test showed strong evidence (χ^2^ = 32.997, *p* < 0.0001, df = 6) linking age categories and depression symptoms. The elder participants in this sample (20–21 years), were more likely (23%) to have severe depression in comparison with 16–19 (12.5%) and 9–15 (9%) years old. Conversely, the younger participants grouped between 9 and 15 (39%) and 20–21 (44%), were less likely to have no depression symptoms in comparison to the 16–19-year-olds (49%). We conducted similar tests to determine the associations between gender, age, anxiety-related disorders (SRQ-20), and sleeping problems and anxiety dreams (NITE). The chi-square test was significant (χ^2^ (1) = 50.992, *p* < 0.0001) to support the association between gender and anxiety-related disorders (SRQ-20). Male participants were less likely (27.9%) to have anxiety-related disorders in comparison to female participants (52.7%). Furthermore, the chi-square test to determine the associations between gender and sleeping difficulties and anxiety dreams (using NITE), showed significant results (χ^2^ (2) = 22.876, *p* < 0.0001). This association revealed that female participants were more likely to have severe nightmare symptoms (43.4%) in comparison to males (32.1%). In turn, male participants were more likely (34.2%) to have some nightmare symptoms present in comparison to female participants (19.5%). The chi-square test to establish the association between age category and anxiety-related disorders (SRQ-20 category) showed moderate evidence (χ^2^ (2) = 7.789, *p* = 0.02). Participants who are 9–15 years (44%) and 20–21 years (46%) were more likely to display anxiety-related symptoms in comparison to 16–19-year-olds (35%). Additionally, there was slight to no evidence of an association between age category and anxiety dreams (NITE category) (χ^2^ (4) = 9.139, *p* = 0.058).

**Table 3:** Associations of gender, age, risk factors and depression.

Variables	*N* = 794		Depression (BDI-II)				Chi-square (χ^2^) (*p*-value)
	Frequency	Percentage (%)	No (45.8%)	Mild (22.2%)	Moderate (17.8%)	Severe (14.2%)	
Gender							
Male	430	54.2%	215 (50.0%)	95 (22.1%)	81 (18.8%)	39 (9.1%)	21.713 (< 0.0001)
Female	364	45.8%	149 (40.9%)	81 (22.3%)	60 (16.5%)	74 (20.3%)	
Age							
G1: 9–15	158	19.9%	61 (38.6%)	37 (23.4%)	64 (29.1%)	14 (8.9%)	32.997 (< 0.0001)
G2: 16–19	455	57.3%	159 (49.0%)	142 (23.1%)	154 (15.4%)	57 (12.5%)	
G3: 20–21	181	22.8%	64 (44.2%)	39 (18.8%)	78 (13.8%)	42 (23.2%)	
Overall family risk							
None	48	6.0%	36 (75.0%)	8 (16.7%)	3 (6.3%)	1 (2.1%)	150.227 (< 0.0001)
Minimal	158	19.9%	99 (62.7%)	32 (20.3%)	23 (14.6%)	4 (2.5%)	
Moderate	182	22.9%	91 (50.0%)	43 (23.6%)	20 (11.0%)	28 (15.4%)	
Worrisome	135	17.0%	52 (38.5%)	28 (20.7%)	41 (30.4%)	14 (10.4%)	
Severe	99	12.5%	17 (17.2%)	21 (21.2%)	17 (17.2%)	44 (44.4%)	
Extreme	172	21.7%	69 (40.1%)	44 (25.6%)	37 (21.5%)	22 (12.8%)	
Overall community risk							
No	121	15.2%	79 (65.3%)	25 (20.7%)	15 (12.4%)	2 (1.7%)	83.131 (< 0.0001)
Minimal	39	4.9%	15 (38.5%)	10 (25.6%)	12 (30.8%)	2 (5.1%)	
Moderate	363	45.7%	174 (47.9%)	87 (24.0%)	66 (18.2%)	36 (9.9%)	
Worrisome	167	21.0%	70 (41.9%)	28 (16.8%)	26 (15.6%)	43 (25.7%)	
Severe	104	13.1%	26 (25.0%)	26 (25.0%)	22 (21.2%)	30 (28.8%)	
Overall school risk							
No	348	43.8%	198 (56.9%)	77 (22.1%)	41 (11.8%)	32 (9.2%)	67.934 (< 0.0001)
Minimal	143	18.0%	54 (37.8%)	38 (26.6%)	21 (14.7%)	30 (21.0%)	
Moderate	64	8.1%	27 (42.2%)	16 (25.0%)	19 (29.7%)	2 (3.1%)	
Worrisome	44	5.5%	12 (27.3%)	6 (13.6%)	13 (29.5%)	13 (29.5%)	
Severe	195	24.6%	73 (37.4%)	39 (20.0%)	47 (24.1%)	36 (18.5%)	

Table 3 also shows the results of chi-square test to check the associations between risks in the family, community, and school and depression (DBI-II). The chi-square test revealed an association between risk in the family space and depression ((χ^2^ (15) = 150.227, *p* < 0.0001). Those with severe risk in the family were more likely to have severe depression issues (around 44.4%), whereas those with no home risk were most likely to have no depression symptoms (75%). The analysis also showed an association between community risk and depression ((χ^2^ (12) = 83.131, *p* < 0.0001). Those with no community risk were most likely to have no depression symptoms (65.3%), whereas those with severe community risk have an even split between each of the categories. In relation to the association between risk at school and depression, the results showed an association between school violence and depression (χ^2^ (12) = 67.934, *p* < 0.0001).

Furthermore, we extended the analysis of the relations between risk, anxiety-related disorders (SRQ) and sleeping problems and anxiety dreams (NITE). The results showed that participants with no or minimal home risk were most likely to have no anxiety-related disorders (87.5% / 82.3%). In contrast, the participants with severe or extreme risk in the family space were more likely to have anxiety-related disorders (70.7% / 58.1%). There was also an association between community risk and anxiety-related disorders ((χ^2^ (4) = 113.35, *p* < 0.0001). Those with severe community risk were more likely to have anxiety-related disorders (70.7%), whereas those with no community violence were most likely to have no anxiety-related disorders (87.5%). The association was also significant in relation to risk factors in school and anxiety-related disorders ((χ^2^ (4) = 77.277, *p* < 0.0001), in that those with no school risks were most likely to have no anxiety-related disorders (74.7%). Finally, the analysis of risk factors and sleeping problems and anxiety dreams revealed that those with severe or extreme family risks were more likely to have severe nightmare issues (around 60%) whereas those participants with no family risks were most likely to have no nightmare symptoms (64.6%). Equally significant was the association between community risks and sleeping problems and anxiety dreams (χ^2^ (8) = 125.668, *p* < 0.0001). Those with severe community risks were more likely to have severe sleeping problems and nightmare issues (around 69.2%), whereas those with no community violence were most likely to have no sleeping problems and nightmare symptoms (51.2%). The association was also significant in relation to school risk factors and sleeping problems and anxiety dreams ((χ^2^ (8) = 140.597, *p* < 0.0001). Those with no school risk are most likely to have no anxiety dreams (50.6%).

### Relation between age, gender, risk and protective factors and odds ratios

Tables 4 and 5, and 6 show the results of a binomial logistic regression to ascertain the effects of age, gender, risk factors [overall family, school, and community risk] and protective factors [siblings, study/work, live with mother and father, parents live together, going to church, having radio] have on the likelihood that participants display mental health problems. The logistic regression model was statistically significant for all three assessments of mental health problems: Depression (BDI-II: (χ^2^(25) = 132.545, *p* < 0.0001), anxiety-related disorders (SQR: (χ^2^(25) = 280.412, *p* < 0.0001), and sleeping problems and anxiety dreams (NITE: (χ^2^(25) = 185.673, *p* < 0.0001)). The model for SQR (Table 4) explained 40.3% (Nagelkerke R^2^) of the variance in anxiety-related disorders and correctly classified 79.3% of cases, likewise for anxiety dreams (NITE) (Table 6) explaining 28.9% of the variance and correctly classifying 71.5% of cases. However, for depression (BDI-II) (Table 5), the model only explained 20.7% of the variance and correctly classified only 64.9% of cases.

**Table 4:** Logistic regression model predictors of anxiety-related disorders (SRQ).

Covariate	*N*	Odds ratio (OR)	95% confidence interval (OR)	*p*-value
Gender				
Male	430	(Ref)	(Ref)	(Ref)
Female	364	0.925	(0.562, 1.521)	0.758
Age	794	0.978	(0.918, 1.042)	0.493
Overall family risk				< 0.0001
None	48	(Ref)	(Ref)	(Ref)
Minimal	158	1.589	(0.565, 4.468)	0.380
Moderate	182	1.640	(0.589, 4.570)	0.344
Worrisome	135	3.223	(1.152, 9.015)	0.026
Severe	99	13.139	(4.445, 38.836)	0.000
Extreme	172	4.420	(1.558, 12.539)	0.005
Overall community risk				< 0.0001
No	121	(Ref)	(Ref)	(Ref)
Minimal	39	2.269	(0.905, 5.689)	0.081
Moderate	363	1.820	(0.987, 3.357)	0.055
Worrisome	167	6.871	(3.134, 15.062)	0.000
Severe	104	2.803	(1.218, 6.451)	0.015
Overall school risk				0.02
No	348	(Ref)	(Ref)	(Ref)
Minimal	143	1.435	(0.848, 2.427)	0.178
Moderate	64	2.643	(1.362, 5.129)	0.004
Worrisome	44	1.452	(0.624, 3.379)	0.387
Severe	195	2.071	(1.154, 3.715)	0.015
Protective factors				
Having siblings				
4 or less		(Ref)	(Ref)	(Ref)
5 or more		1.017	(0.692, 1.494)	0.933
Participation in work force				
No		(Ref)	(Ref)	(Ref)
Yes		3.173	(1.815, 5.547)	< 0.0001
Parents live together				
No	354	(Ref)	(Ref)	(Ref)
Yes	433	0.817	(0.533, 1.252)	0.354
Participation in church				
No		(Ref)	(Ref)	(Ref)
Yes		2.105	(1.387, 3.195)	< 0.0001
Possession of radio				
No		(Ref)	(Ref)	(Ref)
Yes		0.774	(0.533, 1.126)	0.180
Live with				< 0.0001
Alone	37	(Ref)	(Ref)	(Ref)
Mother and father	217	0.336	(0.132, 0.853)	0.022
Father	9	1.086	(0.185, 6.388)	0.927
Mother	95	0.646	(0.234, 1.779)	0.398
Partner	164	1.587	(0.641, 3.925)	0.318
Other	272	0.595	(0.241, 1.467)	0.259

**Table 5:** Logistic regression model predictors of depression (DBI-II).

Covariate	*N*	Odds ratio (OR)	95% confidence interval (OR)	*p*-value
Gender				
Male	430	(Ref)	(Ref)	(Ref)
Female	364	0.741	(0.480, 1.145)	0.741
Age	794	0.974	(0.918, 1.034)	0.392
Overall family risk				< 0.0001
None	48	(Ref)	(Ref)	(Ref)
Minimal	158	1.519	(0.709, 3.253)	0.282
Moderate	182	2.410	(1.124, 5.168)	0.024
Worrisome	135	3.271	(1.474, 7.260)	0.004
Severe	99	9.389	(3.795, 23.232)	0.000
Extreme	172	3.409	(1.501, 7.743)	0.003
Overall community risk				0.001
No	121	(Ref)	(Ref)	(Ref)
Minimal	39	2.294	(1.030, 5.108)	0.042
Moderate	363	1.848	(1.161, 2.941)	0.010
Worrisome	167	1.899	(0.974, 3.702)	0.060
Severe	104	4.454	(2.128, 9.323)	0.000
Overall school risk				0.06
No	348	(Ref)	(Ref)	(Ref)
Minimal	143	1.583	(1.008, 2.488)	0.046
Moderate	64	1.824	(1.020, 3.264)	0.043
Worrisome	44	2.252	(1.026, 4.944)	0.043
Severe	195	1.465	(0.847, 2.532)	0.172
Protective factors				
Having siblings				
4 or less		(Ref)	(Ref)	(Ref)
5 or more		0.985	(0.705, 1.374)	0.927
Participation in work force				
No		(Ref)	(Ref)	(Ref)
Yes		2.004	(1.183, 3.397)	0.010
Parents live together				
No	354	(Ref)	(Ref)	(Ref)
Yes	433	0.715	(0.489, 1.044)	0.082
Participation in church				
No		(Ref)	(Ref)	(Ref)
Yes		1.213	(0.857, 1.716)	0.276
Possession of radio				
No		(Ref)	(Ref)	(Ref)
Yes		0.871	(0.628, 1.209)	0.410 0.692
Live with				
Alone	37	(Ref)	(Ref)	(Ref)
Mother and Father	217	0.500	(0.207, 1.210)	0.124
Father	9	0.900	(0.166, 4.870)	0.903
Mother	95	0.633	(0.241, 1.663)	0.353
Partner	164	0.627	(0.257, 1.532)	0.306
Other	272	0.557	(0.235, 1.321)	0.184

**Table 6:** Logistic regression model predictors of sleeping problems and anxiety dreams (NITE).

Covariate	*N*	Odds ratio (OR)	95% confidence interval (OR)	*p*-value
Gender				
Male	430	(Ref)	(Ref)	(Ref)
Female	364	0.231	(0.140, 0.381)	0.000
Age	794	1.055	(0.989, 1.125)	0.875
Overall family risk				< 0.0001
None	48	(Ref)	(Ref)	(Ref)
Minimal	158	1.951	(0.945, 4.025)	0.071
Moderate	182	3.128	(1.494, 6.550)	0.002
Worrisome	135	4.960	(2.221, 11.077)	0.000
Severe	99	8.616	(3.480, 21.332)	0.000
Extreme	172	7.177	(3.103, 16.596)	0.000
Overall community risk				< 0.0001
No	121	(Ref)	(Ref)	(Ref)
Minimal	39	2.294	(0.920, 5.720)	0.075
Moderate	363	1.277	(0.800, 2.038)	0.306
Worrisome	167	3.554	(1.694, 7.453)	0.001
Severe	104	6.403	(2.857, 14.351)	0.000
Overall school risk				0.01
No	348	(Ref)	(Ref)	(Ref)
Minimal	143	1.926	(1.189, 3.118)	0.008
Moderate	64	2.115	(1.121, 3.992)	0.021
Worrisome	44	2.775	(1.151, 6.695)	0.023
Severe	195	2.762	(1.471, 5.189)	0.002
Protective factors				
Having siblings				
4 or less		(Ref)	(Ref)	(Ref)
5 or more		1.029	(0.717, 1.479)	0.975
Participation in work force				
No		(Ref)	(Ref)	(Ref)
Yes		1.752	(0.962, 3.192)	0.067
Parents live together				
No	354	(Ref)	(Ref)	(Ref)
Yes	433	1.090	(0.720, 1.649)	0.683
Participation in church				
No		(Ref)	(Ref)	(Ref)
Yes		1.477	(1.021, 2.135)	0.038
Possession of radio				
No		(Ref)	(Ref)	(Ref)
Yes		1.480	(1.040, 2.106)	0.029
Live with				0.218
Alone	37	(Ref)	(Ref)	(Ref)
Mother and father	217	0.343	(0.122, 0.960)	0.042
Father	9	0.365	(0.060, 2.234)	0.276
Mother	95	0.563	(0.186, 1.710)	0.311
Partner	164	0.368	(0.131, 1.034)	0.058
Other	272	0.522	(0.190,1.432)	0.207

For SQR, family risk (*p* < 0.0001), community risk (*p* < 0.0001), school risk (*p* = 0.02), and protective factors [study (*p* < 0.0001), church (*p* < 0.0001), live with (*p* < 0.0001)] added significantly to the model/prediction (Table 4), whereas for BDI-II (Table 5) only family risk (*p* < 0.0001), community risk (*p* < 0.0001) and protective factor study/work (*p* = 0.01) were the only factors that added significantly to the prediction of the presence of depression. For sleeping problems and anxiety dreams (Table 6), gender (*p* < 0.0001), community risk (*p* < 0.0001), home risk (*p* < 0.0001) and school risk (*p* = 0.001), as well as protective factors [church (*p* = 0.038) and radio (0.029)] all added significantly to the prediction of whether a youth would be subject to having sleeping problems and anxiety dreams.

The odds of having anxiety-related disorders (SQR: Table 4) is 13.139 times greater for those in the severe family risk category as opposed to those in the no family risk category. The odds of having anxiety-related disorders were 6.871 times greater for those in the worrisome community risk category as opposed to those in the no community risk category. For protective factors, the odds of having anxiety-related disorders were 3.173 times greater for those who do work as opposed to those that do not work and is 2.105 times greater for those who do go to church as opposed to those that did not go to church. Finally, it appears living with parents was best for anxiety-related disorders (SRQ) as the odds of having symptoms are 0.336 times less for those that live with their parents, as opposed to those that live alone.

The odds of having depression (Table 5) were 2.004 times higher for those youth who work compared with those that do not work and 4.454 times higher for those who are in the severe community risk factor group. Like anxiety-related disorders, the odds of having depression were 9.389 times higher in the severe family risk factor group in comparison to those in the no family risk category.

The odds of having anxiety dreams (Table 6) were 0.231 times less for those who are females in comparison with males and 1.477 times higher for those who attend church, compared with those who do not. The odds of having anxiety dreams were 6.503 higher for those who are in the severe community risk factor group and 8.616 higher for those in the severe home risk factor group compared with those who were not suffering these risks.

## Discussion

This article analyzed the frequency and severity of common mental health problems such as depression, anxiety-related disorders, sleeping problems, and anxiety dreams among young people in Mozambique. The country is yet to develop a systematic body of knowledge focused on the mental health needs of young people. While this shortcoming is not unique to Mozambique, Lusophone countries lag when compared to Anglophones in sub-Saharan Africa^59^. This study contributes to breaking on this pattern of regional neglect by involving the perspectives of young people at different stages of the methodology. The findings revealed that gender, age groups, and risk and protective factors influenced the frequency and severity of depression, anxiety-related disorders, sleeping problems, and anxiety dreams of the participants and these had a toll in the lives of young people two decades after the Mozambican civil war’s end. The analysis also showed that risk factors were common in the family, community, and schools. While comparatively with the available published studies, the measuring instruments were different, still the frequency of depression (DBI-II) and anxiety-related disorders (SRQ) was generally consistent with research results which involved subsets of young people in Mozambique such as pregnant women, women with a child under one year and women of childbearing age and without children^33^, young female-headed households^34^, and female victims of intimate partner violence^35^. The frequency of depression and anxiety-related disorders was also consistent with the results of studies conducted in other countries in sub-Saharan Africa^13,14^ and in Europe and the United States^60,61^. However, the severity of depression in this study almost doubled (14.2%) when compared to studies that reported on severity in Ghana (8.1%)^12^ and Tanzania (6%)^15^. The mental health problems of the young people in this study also included sleeping disturbances and anxiety dreams. While sleeping problems have been identified and reported in the existing mental health literature on young people^5,20^, the topic of anxiety dreams remains mostly unexplored. The severity of depression and sleeping disturbances and anxiety dreams among young people in our study might be explained through the impact of poverty and a sense of generalized insecurity as shown by the community risks of “fear of being attacked” (81.6%) and “fear of something” (76.7%). Such a persistent preoccupation and fear, without links to specific events, have been found to increase levels of anxiety sensitivity and depression^62^. The consequences of war trauma, as observed in previous studies in this region^37–43,63^, may have also played a role in the persistence of community fear among the direct survivors of the civil war, which in turn influenced the state of fear among the post-civil war generations and their mental health outcomes in this study. These types of transmission of traumatic memories in the form of persistent fear from one generation to another with detrimental psychological outcomes have been studied as part of the field of intergenerational transmission of traumas. Several studies involving post-Holocaust generations^64–67^, and more recently studies in non-Western settings, have suggested the intergenerational transmission of memories of traumas as drivers of mental health problems among post-civil war and post-genocide young generations^44,68–70^. However, future studies in Mozambique require qualitative and quantitative analysis^71,72^ to systematically examine the linkages and magnitude of intergenerational transmission of war trauma memories.

Gender and age-groups shaped the frequency and severity of depression in this study in slightly different ways. For instance, the associations between gender and mental health outcomes were robust and stable in that across the three measuring instruments the patterns were consistent. In the DBI-II, SRQ, and NITE instruments, girls consistently showed more severe symptoms than boys, which aligns with numerous published studies^16–18^. Our study, however, further suggests an association between gender and the frequency of severe sleeping difficulties and anxiety dreams among young people, with girls reporting more sleeping problems and anxiety dreams than boys. Despite the changes in family relations instigated by the country’s civil war and the recent circulation of mass media technologies, which tends to inspire new models of social relationships, some features of patrilineality have continued which might have wielded serious difficulties on girls and women more than boys and men^30,32,40–43^.

Unlike gender, age-groups showed an unstable and complex relation with mental health outcomes^21,73^, even though the generalized acceptance that most mental disorders have their onset during the adolescence period^22,23^ including the risk of suicides among elementary school-aged children (ages 5–12 years)^24^. In this study, severe depression was more frequent among elder participants (20–21 years) when compared to the younger participants (16–19 years and 9–15 years). This differential outcome based on age-groups is consistent with results from some studies which showed increases with age in the frequency of depressive symptoms^21,60^ and sleeping problems^20^. In the case of our cohort, it is likely that the severity of depression among the age-groups 16–19 and 20–21 was related to the added dosage of risks they experience given the sociocultural expectations and adults’ pressure on them to marry and bearing children. However, in the current context of poverty and unemployment, young people’s attempts to fulfil such obligations might significantly aggravate the already existing burdens in the social lives of these cohorts. Our study also identified two noticeable variations. First, younger participants were more likely than the older participants to have either an increased frequency of mild depression or to have no mild depressive symptoms at all. Second, there were variations in the overall frequency of mental health problems based on the measuring instruments and age-groups in that no strong associations were observed between age-groups and anxiety-related disorders (SRQ) and sleeping problems and anxiety dreams (NITE).

The analysis of risk factors in the family, community, and school and mental health outcomes showed significant associations across the DBI-II, SRQ, and NITE measurements. The more severe the context of risks was, the higher the frequency and severity of depression, anxiety-related disorders, sleeping problems, and anxiety dreams. Other studies have reported on the contributions of risks factors (e.g., poverty, family and community violence, sexual abuse, and bullying in schools) to mental health outcomes^14,15,31,74^. Yet, our study also revealed that risks in the family followed by community risks were the greatest predictors of anxiety-related disorders. In this context, the odds ratio of having anxiety-related disorders was 13.139 times greater for those in the severe family risk category and 6.871 times greater for those in the worrisome community risk category. Overall, the logistic regression analysis to determine the predictive power of the relations between independent variables and outcomes was statistically significant. When we added protective factors into the model, the logistic regression analysis was also statistically significant for all three assessments of mental health problems. Interestingly, while family risk, community risk, school risk, and protective factors [e.g., study, church, live with] added significantly to the predictive model, in turn, age, gender, and a set of other protective factors [siblings, parents live together, radio] did not add significantly to the model. Additionally, the protective factor “live with” [living with mum and dad] was associated with a reduction of anxiety-related disorders in comparison with “living alone” as well as “living with mother or other.” Even if several risks are present in the Gorongosa family environment, the parental presence, and their guardianship appears to be significant sources of protection. The use of the three quantitative assessment instruments provided a robust analysis of the various selected factors and mental health outcomes. Nevertheless, the SRQ appeared the most sensitive diagnostic instrument by correctly classifying 79.3% of anxiety-related cases, followed by NITE correctly classifying 71.5% of cases, and DBI-II which correctly classified 64.9% of cases. This latter result could be explained since depression is multi-faceted and difficult to underpin the underlying issues.

Our study has several limitations. The sample was not nationally representative, and the selection process might have contributed to a bias in our sample. The cross-sectional design and the complexities of validating and applying measuring instruments with participants under 12 years old as well as the time gap between the pilot and full-scale study phases, might have impacted our ability to establish firm conclusions regarding the patterns and durability of mental health problems. Equally the role of culture in shaping the relations between risks and protective factors and how young people conveyed their experiences needs to be more closely analyzed through repeated assessments. This is necessary given the younger age which we work with in these rural communities, and the differences between age as biologically and culturally defined. It was also a limitation that our study was not designed to specifically gauge the long-term impacts of the civil war as drivers of intergenerational transmission of depression, anxiety and sleeping problems and nightmares between parents or other adult guardians and their children.

## Conclusion

This study revealed that gender, age groups, and risk and protective factors influenced the frequency of depression, anxiety-related disorders, sleeping problems, and anxiety dreams of the participants and these had a toll in the lives of young people two decades after the Mozambican civil war’s end. The analysis also showed that risk factors were common in the family, community, and schools. Despite the limitations, our study suggests that understanding the contextual mental health needs of young people in rural Mozambique is crucial for developing insights to craft and implement targeted public mental health interventions in resource-limited settings. Addressing mental health issues among young people necessitates a concentrated effort on understanding and managing the interplay of risk and protective factors within families, schools and communities in resource-limited settings. Furthermore, research in both schools and communities has the advantage of contributing towards reducing stigma around mental health problems among young people and their adult guardians.

## Data Availability

All data generated or analysed during this study are included in this published article and its supplementary information files.

## References

[1] Kutcher, S. et al. Creating evidence-based youth mental health policy in sub-saharan Africa. *Front. Psychiatry*. **10**, 1–8 (2019). 31555156 10.3389/fpsyt.2019.00542 PMC6724683

[2] Patel, V. et al. Mental health of young people. *Lancet*. **369**, 1302–1313 (2007a). 17434406 10.1016/S0140-6736(07)60368-7

[3] Betancourt, T. et al. Sierra Leone’s former child soldiers. *J. Am. Acad. Child. Adolesc. Psychiatry*. **49**, 606–615 (2010). 20494270 10.1016/j.jaac.2010.03.008 PMC3157024

[4] Cliff, J. & Normahomed, A. The impact of war on children’s health in Mozambique. *Soc. Sci. Med.* **36**, 843–848 (1993). 8480230 10.1016/0277-9536(93)90076-g

[5] Fakier, N. & Wild, L. Associations among sleep problems, learning difficulties and substance use in adolescence. *J. Adolescence*. **34**, 717–726 (2011). 10.1016/j.adolescence.2010.09.010 20952052

[6] Jones, L. *Then they Started Shooting: Children of the Bosnian war and the Adults they Become* (Bellevue Literary, 2013).

[7] Klasen, F. et al. Multiple trauma and mental health in former child soldiers. *J. Trauma. Stress*. **23**, 573–581 (2010). 21053376 10.1002/jts.20557

[8] Macksoud, M. & Aber, J. The war experiences and psychosocial development of children in Lebanon. *Child Dev.* **67**, 70–88 (1996). 8605835

[9] Mels, C. et al. The psychological impact of forced displacement and related risk factors in eastern Congolese adolescents affected by war. *J. Child. Psychol. Psychiatry*. **51**, 1096–1104 (2010). 20345839 10.1111/j.1469-7610.2010.02241.x

[10] Neugebauer, R. et al. Post-traumatic stress reactions among Rwandan children and adolescents in the early aftermath of genocide. *Int. J. Epidemiol.* **38**, 1033–1045 (2009). 19204009 10.1093/ije/dyn375

[11] Neuner, F. et al. Prevalence, predictors and outcomes of spirit possession experiences among former child soldiers and war-affected civilians in Northern Uganda. *Soc. Sci. Med.* **75**, 548–554 (2012). 22580073 10.1016/j.socscimed.2012.03.028

[12] Asante, K. & Andoh-Arthur, J. Prevalence and determinants of depressive symptoms among university students in Ghana. *J. Affect. Disorders*. **171**, 161–166 (2015). 25305431 10.1016/j.jad.2014.09.025

[13] Kilburn, K. et al. Examination of performance of the Center for epidemiologic studies Depression Scale Short Form 10 among African youth in poor, rural households. *BMC Psychiatry*. **18**, 1–13 (2018). 29914413 10.1186/s12888-018-1774-z PMC6006708

[14] Nguyen, K. et al. Coerced and forced sexual initiation and its association with negative health outcomes among youth. *Child. Negl. Abuse*. **96**, 1–10 (2019). 10.1016/j.chiabu.2019.104074 PMC6760991 31445403

[15] Kuringe, E. et al. Prevalence and correlates of depression and anxiety symptoms among out-of-school adolescent girls and young women in Tanzania. *PLoS ONE*. **14**, e0221053 (2019). 31419238 10.1371/journal.pone.0221053 PMC6697336

[16] Ferrari, A. J. et al. Burden of depressive disorders by country, sex, age, and year. *PLoS Med.* **10**, 1–12 (2013). 10.1371/journal.pmed.1001547 PMC3818162 24223526

[17] Seedat, S. et al. Cross-national associations between gender and mental disorders in the World Health Organization World mental health surveys. *Arch. Gen. Psychiatry*. **66**, 785–795 (2009). 19581570 10.1001/archgenpsychiatry.2009.36 PMC2810067

[18] Stansfeld, S. et al. Exposure to violence and mental health of adolescents. *BJPsych Open.* **3**, 257–264 (2017). 29093828 10.1192/bjpo.bp.117.004861 PMC5643877

[19] Gbadamosi, I. et al. Depression in Sub-saharan Africa. *IBRO Neurosci. Rep.* **12**, 309–322 (2022). 35746974 10.1016/j.ibneur.2022.03.005 PMC9210463

[20] Ohayon, M. et al. Prevalence and patterns of problematic sleep among older adolescents. *J. Am. Acad. Child. Adolesc. Psychiatry*. **39**, 1549–1556 (2000). 11128333 10.1097/00004583-200012000-00019

[21] Kessler, R. et al. Lifetime prevalence and age-of-onset distributions of mental disorders in the World Health Organization’s World Mental Health Survey Initiative. *World Psychiatry*. **6**, 168–176 (2007). 18188442 PMC2174588

[22] Belfer, M. Child and adolescent mental disorders. *J. Child. Psychol. Psychiatry*. **49**, 226–236 (2008). 18221350 10.1111/j.1469-7610.2007.01855.x

[23] Haney, E. et al. One size does not fit all: psychometric properties of the Shona Symptom Questionnaire (SSQ) among adolescents and young adults in Zimbabwe. *J. Affect. Disorders*. **167**, 358–367 (2014). 25020271 10.1016/j.jad.2014.05.041 PMC4894474

[24] Bridge, J. Suicide trends among elementary school-aged children in the United States from 1993 to 2012. *JAMA Pediatr.* **169**, 673–677 (2015). 25984947 10.1001/jamapediatrics.2015.0465

[25] WHO. *Adolescent Mental Health* (World Health Organization, 2021).

[26] Kim, M. et al. Prevalence of depression and validation of the Beck Depression Inventory-II and the children’s Depression Inventory-Short amongst HIV-positive adolescents in Malawi. *J. Int. AIDS Soc.* **17**, 1–8 (2014). 10.7448/IAS.17.1.18965 PMC4119288 25085002

[27] Devereux, G. *Normal and Abnormal* (Chicago University Press, 1980).

[28] Hawkins, J. D. et al. Risk and protective factors for alcohol and other drug problems in adolescence and early adulthood. *Psychol. Bull.* **112**, 64–105 (1992). 1529040 10.1037/0033-2909.112.1.64

[29] Meinck, F. et al. Risk and protective factors for physical and emotional abuse victimisation amongst vulnerable children in South Africa. *Child Abuse Rev.* **24**, 182–197 (2015).

[30] Wado, Y. D. et al. Pregnancy and early motherhood among adolescents in five east African countries. *BMC Pregnancy Childbirth*. **19**, 1–11 (2019). 30727995 10.1186/s12884-019-2204-z PMC6366026

[31] Glendinning, A. & West, P. Young people’s mental health in context. *Soc. Sci. Med.* **65**, 1180–1191 (2007). 17576031 10.1016/j.socscimed.2007.05.012

[32] Halsted, S. et al. Depressive symptoms, suicidal ideation, and mental health careseeking in central Mozambique. *Soc. Psychiatry Psychiatr. Epidemiol.* **54**, 1519–1533 (2019). 31317245 10.1007/s00127-019-01746-2 PMC7050264

[33] Khan, S. et al. Women’s mental health in Mozambique. *Global Mental Health*. **9**, 38–44 (2022). 36618727 10.1017/gmh.2022.1 PMC9806954

[34] Audet, C. et al. Depression among female heads-of-household in rural Mozambique. *J. Affect. Disorders*. **227**, 48–55 (2018). 29053975 10.1016/j.jad.2017.10.022 PMC5805617

[35] Zacarias, A. et al. Symptoms of depression, anxiety, and somatization in female victims and perpetrators of intimate partner violence in Maputo City, Mozambique. *Int. J. Women’s Health*. **4**, 491–503 (2012). 23071419 10.2147/IJWH.S29427 PMC3469227

[36] Seidu, A-A. et al. Suicidal behaviours among in-school adolescents in Mozambique. *PLoS ONE*. **15**, e0236448 (2020). 32706805 10.1371/journal.pone.0236448 PMC7380623

[37] Igreja., V., Kleijn, W. & Richters, A. Women’s posttraumatic suffering after the war in Mozambique. *J. Nerv. Mental Disease*. **194**, 502–509 (2006). 10.1097/01.nmd.0000228505.36302.a3 16840846

[38] Schreuder, B., Igreja, V., Dijk, J. & Kleijn, W. Intrusive re-experiencing of chronic strife or war. *Adv. Psychiatr. Treat.* **7**, 102–108 (2001).

[39] Igreja, V., Schreuder, B., Dijk, A. & Kleijn, W. Testimony method to ameliorate post-traumatic stress symptoms. *Br. J. Psychiatry*. **184**, 251–257 (2004). 14990524 10.1192/bjp.184.3.251

[40] Deacon, Z. & Sullivan, C. An ecological examination of rural Mozambican women’s attainment of postwar wellbeing. *J. Community Psychol.* **38**, 115–130 (2010).

[41] Wiegink, N. *Former Guerrillas in Mozambique* (University of Pennsylvania, 2020).

[42] Igreja, V. Cultural disruption and the care of infants in post-war Mozambique in *Children and youth on the front line* (eds. Boyden, J. & de Berry, J.) 23–41 (Berghahn, 2004).

[43] Igreja, V., Colaizzi, J. & Brekelmans, A. Legacies of civil wars. *Br. J. Sociol.* **72**, 426–447 (2021). 33368164 10.1111/1468-4446.12802 PMC8247410

[44] Beck, A., Steer, R. & Brown, G. *Manual for the Beck Depression Inventory* (The Psychological Corporation, 1996).

[45] Whisman, M. et al. Factor structure of the Beck Depression Inventory— Second Edition (BDI-II) in a student sample. *J. Clin. Psychol.* **56**, 545–551 (2000). 10775046 10.1002/(sici)1097-4679(200004)56:4<545::aid-jclp7>3.0.co;2-u

[46] Campos, R. & Gonçalves, B. The Portuguese version of the Beck Depression Inventory-II (BDI-II). *Eur. J. Psychol. Assess.* **27**, 258–264 (2011).

[47] Byrne, B. et al. Validating the Beck Depression Inventory-II for Hong Kong community adolescents. *Int. J. Test.* **4**, 199–216 (2004).

[48] Makhubela, M. & Mashegoane, S. Validation of the Beck Depression Inventory–II in South Africa. *South. Afr. J. Psychol.* **46**, 203–221 (2016).

[49] Kortmann, F. & Horn, S. Comprehension and motivation in responses to a psychiatric screening instrument. *Br. J. Psychiatry*. **153**, 95–101 (1988). 3224257 10.1192/bjp.153.1.95

[50] World Health Organization. *A User’s Guide the Self-Reporting Questionnaire (SRQ-20)* (World Health Organization, 1994).

[51] Deshpande, S. et al. Psychiatric disorders among medical in-patients in an Indian hospital. *Br. J. Psychiatry*. **154**, 504–509 (1989). 2590781 10.1192/bjp.154.4.504

[52] Giang, K. et al. The Vietnamese version of the self-reporting questionnaire 20 (SRQ-20) in detecting mental disorders in rural Vietnam. *Int. J. Social Psychiatry*. **52**, 175–184 (2006). 10.1177/0020764006061251 16615249

[53] Scholte, W. et al. Psychometric properties and longitudinal validation of the self-reporting questionnaire (SRQ-20) in a Rwandan community setting. *BMC*. **11**, 1–10 (2011). 10.1186/1471-2288-11-116 PMC3173390 21846391

[54] Igreja, V. et al. Agricultural cycle and the prevalence of posttraumatic stress disorder. *J. Trauma. Stress*. **22**, 172–179 (2009). 19489043 10.1002/jts.20412

[55] Feijó, R., Saueressig, M., Salazar, C. & Chaves, M. Mental health screening by self-report questionnaire among community adolescents in southern Brazil. *J. Adolesc. Health*. **20**, 232–237 (1997). 9069024 10.1016/S1054-139X(96)00085-7

[56] Pinheiro, K. et al. Common mental disorders in adolescents. *Brazilian J. Psychiatry*. **29**, 241–245 (2007). 10.1590/s1516-44462006005000040 17713704

[57] Schreuder, B., van Egmond, M., Kleijn, W. & Visser, A. Daily reports of posttraumatic nightmares and anxiety dreams in Dutch war victims. *J. Anxiety Disorders*. **12**, 511–524 (1998). 10.1016/s0887-6185(98)00032-2 9879032

[58] Reeskamp, H. Working with dreams in a clinical setting. *Am. J. Psychother.* **60**, 23–36 (2006). 16770914 10.1176/appi.psychotherapy.2006.60.1.23

[59] Wittman, L., Zehnder, D., Schredl, M., Jenni, O. & Landolt, M. Posttraumatic nightmares and psychopathology in children after road traffic accidents. *J. Trauma. Stress*. **23**, 232–239 (2010). 20419731 10.1002/jts.20514

[60] Patel, V. et al. Treatment and prevention of mental disorders in low-income and middle-income countries. *Lancet*. **370**, 991–1005 (2007b). 17804058 10.1016/S0140-6736(07)61240-9

[61] Saluja, G. et al. Prevalence of and risk factors for depressive symptoms among young adolescents. *Arch. Pediatr. Adolesc. Med.* **158**, 760–765 (2004). 15289248 10.1001/archpedi.158.8.760

[62] Verhulst, F. et al. The prevalence of DSM-III-R diagnoses in a national sample of Dutch adolescents. *Arch. Gen. Psychiatry*. **54**, 329–336 (1997). 9107149 10.1001/archpsyc.1997.01830160049008

[63] Igreja, V. Dias-Lambranca. The social world of dreams and nightmares in a post-conflict setting. *Intervention*. **4**, 147–159 (2006).

[64] Jemcov, A. et al. Do anxiety sensitivity cognitive concerns and/or depression symptoms independently explain sleep disturbances in a high anxiety sensitive treatment-seeking sample. *J. Anxiety Disorders*. **97**, 1–7 (2023). 10.1016/j.janxdis.2023.102731 37236069

[65] Lehrner, A. & Yehuda, R. Trauma across generations and paths to adaptation and resilience. *Psychol. Trauma.* **10**, 22–29 (2018). 29323523 10.1037/tra0000302

[66] Lev-Wiesel, R. Intergenerational transmission of trauma across three generations. *Qualitative Social Work*. **6**, 75–94 (2007).

[67] Scharf, M. Long-term effects of trauma. *Dev. Psychopathol.* **19**, 603–622 (2007). 17459186 10.1017/S0954579407070290

[68] Wiseman, H. et al. Parental communication of Holocaust experiences and interpersonal patterns in offspring of Holocaust survivors. *Int. J. Behav. Dev.* **26**, 371–381 (2002).

[69] Roth, M., Neuner, F. & Elbert, T. Transgenerational consequences of PTSD: risk factors for the mental health of children whose mothers have been exposed to the Rwandan genocide. *Int. J. Mental Health Syst.* **8**, 1–12 (2014). 10.1186/1752-4458-8-12 PMC3978019 24690436

[70] Münyas, B. Genocide in the minds of Cambodian youth: transmitting (hi)stories of genocide to second and third generations in Cambodia. *J. Genocide Res.* **10**, 413–439 (2008).

[71] Igreja, V. & E. Baines. Social trauma and recovery in *A companion to the anthropology of Africa* (eds Grinker, R. et al. Lubkemann, S., Steiner, C. & Gonçalves, E.) 249 – 270 (Wiley, 2019).

[72] Igreja, V. Relationships in transition and negotiating silence in Mozambique in Truth, Silence and Violence in Emerging States (ed Russell, A.) 88–110 (Routledge, 2018).

[73] Barron, I. & Abdallah, G. Intergenerational trauma in the occupied Palestinian territories: effect on children and promotion of healing. *J. Child. Adolesc. Trauma.* **8**, 103–110 (2015).

[74] Rothon, C. et al. Can social support protect bullied adolescents from adverse outcomes? *J. Adolescence*. **34**, 579–588 (2011). 10.1016/j.adolescence.2010.02.007 PMC3107432 20637501

